# Bilateral Cavernous Sinus Thrombosis and Clival Osteomyelitis Complicating Pediatric Otitis Media

**DOI:** 10.7759/cureus.111836

**Published:** 2026-06-30

**Authors:** Amal Akammar, Hajar Ouazzani Chahdi, Ismail Chaouche, Nizar El Bouardi, Meriem Haloua, Badreeddine Alami, Youssef Alaoui Lamrani, Mustapha Maaroufi, Meryem Boubbou

**Affiliations:** 1 Department of Maternal and Child Radiology, Hassan II University Hospital, Faculty of Medicine, Pharmacy and Dentistry of Fez, Sidi Mohamed Ben Abdellah University, Fez, MAR; 2 Department of Radiology, Hassan II University Hospital, Faculty of Medicine, Pharmacy and Dentistry of Fez, Sidi Mohamed Ben Abdellah University, Fez, MAR

**Keywords:** acute otitis media, cavernous sinus thrombosis, central skull base osteomyelitis, cerebral venous and dural sinus thrombosis, pediatric skull base osteomyelitis

## Abstract

Central skull base osteomyelitis (SBO) and associated neurovascular complications represent critical clinical conditions that necessitate prompt diagnosis and aggressive therapeutic intervention. We report the case of a previously healthy four-year-old boy who presented with a six-day history of purulent left otorrhea, fever, headaches, and the acute onset of left convergent strabismus. Contrast-enhanced CT revealed left-sided acute otitis media complicated by bilateral cavernous sinus thrombosis, right ophthalmic vein thrombosis, left sigmoid sinus and internal jugular vein thrombosis, and an associated subperiosteal orbital abscess. Concurrently, extensive clival osteolysis confirmed a diagnosis of central SBO. The patient was successfully managed with an aggressive therapeutic protocol comprising targeted high-dose antibiotics and anticoagulation, leading to complete clinical and laboratory recovery. This case emphasizes the necessity of immediate advanced neuroimaging when cranial nerve deficits accompany a routine pediatric ear infection and for prompt initiation of dual antimicrobial and anticoagulant therapy to avert catastrophic outcomes.

## Introduction

Skull base osteomyelitis (SBO) is an aggressive, necrotizing infection affecting the osseous structures of the cranial base, including the temporal, sphenoid, and occipital bones [1]. Classically, SBO presents as “typical SBO,” arising from necrotizing external otitis in elderly diabetic or immunocompromised individuals, with *Pseudomonas aeruginosa* as the predominant causative pathogen [1-3]. Conversely, “atypical” or central SBO originates within the sphenoid or occipital bones, often in the absence of external otitis, secondary to paranasal sinusitis, dental infections, or hematogenous dissemination [4].

Regardless of classification, SBO is extremely rare in the pediatric population because of the high vascularity of the young diploic bone and the widespread use of empiric antibiotic therapy for childhood ear and sinus infections [5]. When it does occur in children, it is typically an insidious complication of acute otitis media (AOM) or sinusitis [1,2,6]. The pediatric skull base contains a complex network of interconnecting vascular channels, unossified synchondroses, and cranial foramina that can facilitate the rapid, unrestricted spread of infection to adjacent compartments [5,6]. Consequently, pediatric SBO can precipitate devastating intracranial and vascular complications, including meningitis, intracranial empyema, carotid endarteritis, mycotic aneurysms, and extensive dural venous sinus thrombosis [6,7].

Because the clinical presentation in children often mimics skull base malignancies, paraclival masses, or benign cranial neuropathies, diagnostic delays are common [7,8]. Clinicians often face a diagnostic dilemma distinguishing SBO from rhabdomyosarcoma, lymphoma, or Langerhans cell histiocytosis [8]. Advanced radiological evaluation plays a critical role in overcoming this dilemma, where high-resolution CT and contrast-enhanced MRI serve complementary roles to precisely delineate cortical destruction, bone marrow infiltration, and extensive vascular involvement [1,2,9].

The primary objective of this case report is to highlight a rare and aggressive presentation of atypical pediatric central SBO and multi-sinus dural venous thrombosis following AOM, detailing the diagnostic hurdles and the successful multi-modal management strategy.

## Case presentation

A previously healthy four-year-old boy presented to our pediatric emergency department with a six-day history of left-sided purulent otorrhea, headaches, and cough in a febrile context. He had been initially managed at a regional hospital, where he received intravenous ceftriaxone, gentamicin, and metronidazole. Laboratory investigations at that time demonstrated a severe inflammatory syndrome. During that admission, the sudden onset of convergent strabismus was noted, prompting his immediate transfer to our tertiary university hospital for specialized, multidisciplinary management (Figure 1).

**Figure 1:**
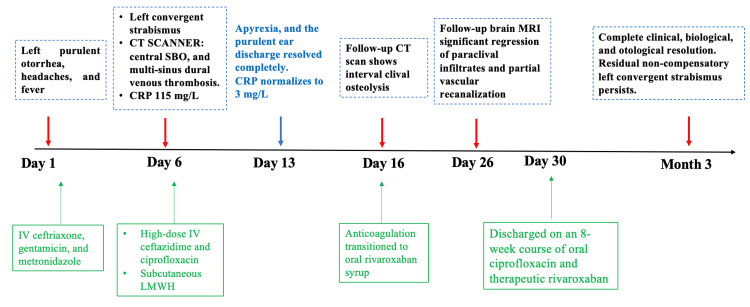
Timeline of patient clinical course, radiological evolution, and therapeutic management CT: computed tomography, SBO: skull base osteomyelitis, CRP: C-reactive protein, MRI: magnetic resonance imaging, IV: intravenous, LMWH: low-molecular-weight heparin

On admission, his vital signs were temperature 38.7°C, heart rate 110 beats per minute, respiratory rate 28 breaths per minute, and blood pressure 124/77 mmHg. Physical examination revealed a conscious but highly agitated child. Ear, nose, and throat evaluation showed abundant, thick purulent secretions within the left external auditory canal, alongside significant congestion of the tympanic membrane.

Neurological assessment revealed an isolated, prominent left-sided convergent strabismus, consistent with an uncompensated left abducens nerve (CN VI) palsy. Bilateral pupillary light reflexes were intact and symmetric, and the remaining cranial nerve examination was unremarkable. There were no gross motor or sensory focal deficits. Deep tendon reflexes were normal, and meningeal signs were absent. An ophthalmic evaluation under sedation confirmed isolated right upper eyelid edema, right-sided limitation of abduction, and mild enophthalmos.

An initial contrast-enhanced brain and maxillofacial CT scan performed upon transfer showed findings consistent with acute left-sided otitis media. Additionally, a hypodense infiltrate with delayed contrast enhancement was identified involving the bilateral cavernous sinuses, extending anteriorly to the orbital apices and posteriorly to the retroclival region and retropharyngeal space through the foramen lacerum. At this level, a small rim-enhancing hypodense collection was noted, associated with extensive soft tissue infiltration of the left infratemporal region. Bilateral absence of cavernous sinus opacification was observed, extending to the right ophthalmic vein, confirming bilateral cavernous sinus thrombosis. The intracavernous segments of the internal carotid arteries demonstrated bilateral luminal narrowing with wall thickening, suggestive of carotid endarteritis. A focal opacification defect of the left sigmoid sinus and the internal jugular vein distal to the jugular bulb, which remained well opacified, indicated focal venous thrombosis at these levels. Finally, a small, septated intraconal collection measuring 13 × 14 mm was noted adjacent to the medial rectus muscle, showing peripheral rim enhancement following contrast administration, consistent with a subperiosteal orbital abscess (Figure 2).

**Figure 2:**
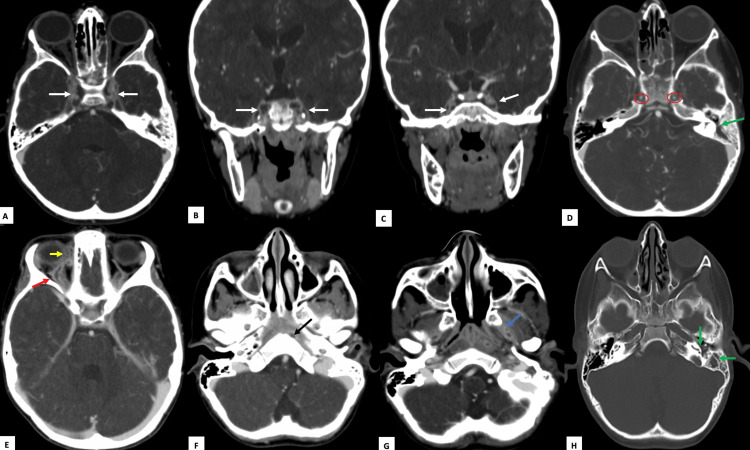
Contrast-enhanced brain and maxillofacial CT scan (axial sections A-E, coronal sections F-G, and axial bone window section H) demonstrating the initial imaging findings (A, B, and C) Axial and coronal contrast-enhanced CT sections at the level of the cavernous sinuses showing bilateral hypodense infiltration with absent contrast opacification (white arrows), indicative of bilateral cavernous sinus thrombosis. (D) Axial contrast-enhanced CT section demonstrating fluid opacification of the left middle ear cavity and left mastoid air cells (green arrows), consistent with acute left-sided otitis media. Concurrently, bilateral wall thickening and luminal narrowing of the intracavernous segments of the internal carotid arteries are visible (red circles), secondary to septic carotid endarteritis from the adjacent paraclival infectious process. (E) Axial contrast-enhanced CT section at the orbital level demonstrating thrombosis of the right ophthalmic vein (red arrow) associated with a right extraconal collection (yellow arrow), reflecting contiguous spread of the infection into the orbital compartment. (F and G) Coronal contrast-enhanced CT sections at the level of the sphenoid sinus demonstrating a small rim-enhancing hypodense collection within the retropharyngeal and pre-sphenoidal spaces (black arrow), associated with extensive infiltration of the deep cervical spaces extending into the left infratemporal fossa (blue arrow). (H) Axial bone window CT section confirming the absence of frank osseous lysis at this early stage, with no identifiable cortical destruction or trabecular disruption of the clivus, alongside fluid opacification of the left middle ear cavity and mastoid air cells (green arrows). CT: computed tomography

Initial laboratory investigations at our center confirmed significant systemic inflammation, with a CRP of 115 mg/L and a white blood cell count of 19,830/μL. Baseline hematological, renal, and hepatic profiles were performed to assess organ function prior to treatment initiation (Table 1).

**Table 1:** Initial blood laboratory parameters WBC: white blood cell count, CRP: C-reactive protein, ALT: alanine aminotransferase, AST: aspartate aminotransferase

Parameter	Patient value	Normal range and units	Interpretation/clinical significance
WBC	19,830	5,000-12,000 /μL	Elevated: indicates acute systemic infection
Neutrophils	15,650	1,500-8,500 /μL	Elevated
Hemoglobin	10	11.0-14.0 g/dL	Mild anemia
Platelets	744,000	150,000-450,000 /μL	Marked thrombocytosis
CRP	115	<5.0 mg/L	Significantly elevated
ALT	22	10-35 U/L	Normal
AST	28	15-40 U/L	Normal
Creatinine	0.38	0.30-0.70 mg/dL	Normal
Blood urea nitrogen	11	7-20 mg/dL	Normal

A diagnostic lumbar puncture was performed to rule out contiguous acute bacterial meningitis. Analysis of cerebrospinal fluid (CSF) alongside extensive serum immunologic testing confirmed the absence of primary or secondary immunodeficiency (Table 2). CSF cultures and polymerase chain reaction (PCR) testing on the GeneXpert® System (Cepheid, Sunnyvale, CA, USA) were negative. Repeated cultures of the purulent ear discharge remained sterile, likely due to the prior antibiotic therapy.

**Table 2:** CSF analysis and immunological workup CSF: cerebrospinal fluid, WBC: white blood cell count, PMN: polymorphonuclear leukocytes, PCR: polymerase chain reaction, HIV: human immunodeficiency virus, CH50: total hemolytic complement, IgG: immunoglobulin G, IgA: immunoglobulin A, IgM: immunoglobulin M

Diagnostic test	Patient value	Normal range	Interpretation/clinical significance
CSF color	Clear	Clear	Normal
CSF WBC	13	0-5/μL	Mild pleocytosis
CSF PMN %	11%	0%	Mildly elevated
CSF lymph %	89%	60-100%	Normal
CSF glucose	51	40-70 mg/dL	Normal
CSF protein	45	15-45 mg/dL	Normal
CSF culture	Sterile	Sterile	Negative
CSF PCR	Negative	Negative	Negative
HIV screening	Non-reactive	Non-reactive	Negative
Complement CH50​	42	30-60 U/mL	Normal
Serum IgG	840	450-1,150 mg/dL	Normal
Serum IgA	92	20-100 mg/dL	Normal
Serum IgM	112	40-160 mg/dL	Normal

Given the severity of the combined central SBO and multi-sinus dural venous thrombosis, an aggressive therapeutic protocol was initiated. Empiric antibiotic coverage was escalated to high-dose intravenous ceftazidime (150 mg/kg/day in three divided doses) and ciprofloxacin (30 mg/kg/day in two divided doses) to achieve optimal penetration into bone and the central nervous system. Anticoagulation was simultaneously started with low-molecular-weight heparin (enoxaparin, 1,500 IU every 12 hours subcutaneously) and subsequently transitioned to oral rivaroxaban syrup upon clinical stabilization.

A follow-up contrast-enhanced cerebral CT performed ten days after the initial imaging revealed persistence of the hypodense infiltrate within the bilateral cavernous sinuses, with continued extension anteriorly to the orbital apices and posteriorly to the retroclival region and retropharyngeal space. Compared with the first scan, interval progression was observed, with new involvement of the left hemi-sphenoid sinus and a heterogeneous lytic appearance of the clivus, confirming the diagnosis of central SBO (Figure 3).

**Figure 3:**
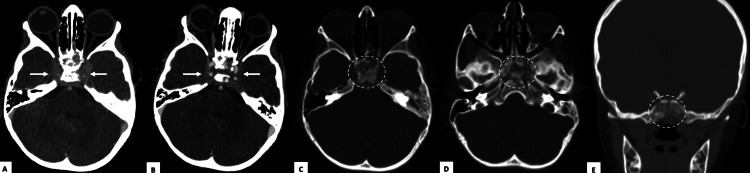
Follow-up contrast-enhanced brain CT scan performed ten days after the initial imaging, showing interval progression of the infectious process (A, B) Axial contrast-enhanced CT sections (parenchymal window) demonstrating persistence of the hypodense infiltrate within the bilateral cavernous sinuses, with continued absence of contrast opacification (white arrows). (C, D, E) Axial bone window CT sections demonstrating the development of frank osseous lysis, characterized by a heterogeneous and disrupted trabecular pattern involving the clivus and sphenoid bone (white circles). CT: computed tomography

The patient responded favorably to this strategy. He achieved sustained apyrexia within the first week, and the purulent ear discharge resolved completely. Follow-up laboratory evaluation demonstrated a normalized white blood cell count (9,360/μL) and a marked decrease in inflammatory markers, with the CRP dropping from 115 mg/L to 3 mg/L.

A follow-up brain MRI obtained 20 days after treatment initiation demonstrated significant radiological improvement. There was marked regression of the hypodense infiltrative process within the cavernous sinuses, orbital apices, and retroclival and retropharyngeal spaces, alongside partial recanalization of the right sigmoid sinus and internal jugular vein (Figure 4).

**Figure 4:**
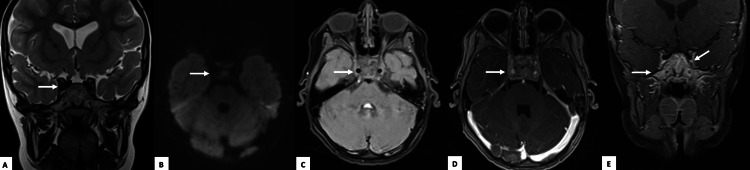
Follow-up brain MRI 20 days post-treatment (A: coronal T2, B: DWI, C: unenhanced axial T1, D: contrast-enhanced axial T1, E: contrast-enhanced coronal T1), demonstrating marked radiological resolution All sequences show significant regression of the paraclival and bilateral cavernous sinus infiltrate (white arrows). Key improvements include partial restoration of normal fatty clival bone marrow signal on unenhanced T1 (C), absence of restricted diffusion on DWI (B), ruling out residual abscess, and near-complete resolution of pathological contrast enhancement (D, E), confirming a favorable response to therapy. MRI: magnetic resonance imaging, DWI: diffusion-weighted imaging

The patient was safely discharged to complete an eight-week course of oral ciprofloxacin and therapeutic rivaroxaban. At his three-month outpatient evaluation, the child was in excellent general health with completely normal otoscopic findings. However, a residual non-compensatory convergent strabismus persisted, requiring ongoing pediatric ophthalmology follow-up and planning for corrective strabismus surgery.

## Discussion

SBO remains an exceptionally rare and perilous diagnosis in pediatrics, with its exact incidence poorly defined due to a literature that relies heavily on isolated case reports and small series [1,2,6]. Systematic reviews and multicenter series indicate that children account for fewer than 20% of all documented SBO cases, with central variants involving the clivus and sphenoid bone representing particularly exceptional clinical entities [5-7]. A distinct male predominance and a higher vulnerability in children under five have been consistently reported across published series, a demographic profile that precisely mirrors our patient [6,10]. Unlike adult SBO, which tracks almost exclusively with elderly, diabetic, or otherwise immunocompromised patients, the pediatric form routinely strikes previously healthy, immunocompetent children, frequently disarming early clinical suspicion [1,3,6].

The pathophysiology of pediatric SBO differs substantially from the classic adult model. In adults, SBO is almost universally an otogenic complication, driven by *Pseudomonas aeruginosa*, that exploits the compact Haversian system of the temporal bone through the fissures of Santorini [1,3]. Conversely, pediatric central SBO typically exploits atypical, non-otogenic pathways. In early childhood, AOM or sinusitis spreads via retrograde thrombophlebitis through the valveless diploic veins or via direct contiguous extension across the unossified skull base synchondroses, tracking straight to the sphenoid body and clivus [2,5,6]. This case closely mirrors such a trajectory: a routine episode of left-sided AOM in an immunocompetent four-year-old ignited a massive infectious cascade. The process migrated anteromedially, breaching the sphenoid sinus and clivus, while simultaneously triggering septic thrombophlebitis of multiple local dural venous structures [5,6].

Bilateral cavernous sinus thrombosis represents a catastrophic endpoint for these deep cranial infections [5]. The immediate proximity of the cavernous sinus to both the orbital apex and the central skull base explains why our patient initially presented with convergent strabismus and right orbital apex syndrome. Because the abducens nerve (CN VI) runs unprotected through the center of the cavernous sinus next to the internal carotid artery, it is uniquely vulnerable to compressive ischemia or infectious endarteritis from paraclival phlegmons [5,6]. Furthermore, the concomitant thrombosis of the left sigmoid sinus and internal jugular vein highlights how aggressively pediatric central SBO can drive multifocal venous complications through contiguous septic spread [6,10].

From a diagnostic standpoint, central SBO is highly deceptive. Its clinical footprint (persistent headaches, low-grade pyrexia, and cranial nerve palsies) frequently mimics pediatric skull base malignancies such as rhabdomyosarcoma, lymphoma, or nasopharyngeal carcinoma [5,6,8]. In our case, the diagnostic timeline was protracted by the complete absence of classical risk factors, an initially misleading picture of simple otitis media, and the velocity with which multifocal intracranial complications unfolded. Clinicians must maintain a low threshold for suspecting SBO in any child presenting with a sinogenic or otogenic infection paired with cranial nerve deficits or a systemic inflammatory syndrome that resists standard antibiotic regimens [1,2,10].

Definitive mapping of the central SBO and its vascular sequelae requires a multimodal imaging protocol combining high-resolution CT and contrast-enhanced MRI [1,5,6].

On CT, high-resolution bone window reconstructions are the gold standard for tracking osseous changes [1,5]. The hallmark signatures include cortical demineralization, loss of the normal trabecular architecture of the clivus, and eventual frank osteolysis with cortical fragmentation [5,6]. It is important to note that trabecular bone erosion of 30-50% is required to produce a positive CT finding, meaning that early osseous changes frequently lag behind active soft-tissue infection by several days to weeks, potentially yielding false-negative results in the early disease course [4]. Subacute forms may also display reactive periosteal remodeling and sclerotic changes. Soft-tissue windows are essential to delineate paraclival and retropharyngeal infiltration, obliteration of normal fat planes, and rim-enhancing hypodense fluid collections indicative of abscess formation [1,6]. Contrast-enhanced CT venography effectively flags dural venous sinus thrombosis, often via the classic “delta sign” or complete non-opacification of the cavernous sinuses, and can identify internal carotid artery wall thickening consistent with septic endarteritis [5,6]. In this patient, the initial CT provided decisive diagnostic leverage by capturing clival osteolysis, paraclival soft-tissue infiltration, and extensive venous filling defects despite the early stage of the disease.

Contrast-enhanced MRI with dedicated skull base sequencing remains the gold standard for diagnostic staging and surveillance [1,5,6]. The earliest and most sensitive marker of osteomyelitis is the replacement of normal fatty bone marrow, visible on T1-weighted imaging as a hypointensity within the clivus that matches the T2- and STIR-weighted hyperintensity, reflecting intraosseous edema [2,6]. Post-gadolinium T1-weighted sequences typically demonstrate intense, heterogeneous enhancement of the marrow and adjacent soft tissues, precisely mapping the boundaries of extraosseous spread into the prevertebral, retropharyngeal, and infratemporal spaces [1,5]. DWI provides critical diagnostic utility by demonstrating restricted diffusion within intracranial or orbital abscesses, reliably distinguishing them from sterile reactive fluid collections or neoplastic tissue, and by showing significantly higher apparent diffusion coefficient values in SBO than in lymphoma and nasopharyngeal carcinoma [9]. MR venography offers highly sensitive assessment of dural sinus involvement, detecting subtle, non-occlusive thrombi that CT may miss [6]. The use of fat-suppressed T2 and gadolinium-enhanced T1 fat-suppressed sequences is, furthermore, crucial for detecting early perineural spread, cavernous sinus invasion, and orbital apex involvement, all of which carry significant prognostic implications [5,6]. As noted by Kristenson et al. [3], deep paraclival inflammatory masses can easily mimic malignancy; here, careful MRI analysis ruled out cavum mucosal lesions, safely eliminating nasopharyngeal carcinoma from the differential and sparing the child an invasive biopsy.

Importantly, serial neuroimaging is mandatory in pediatric central SBO. Clinical recovery routinely precedes radiological resolution by several weeks, and stopping therapy prematurely based solely on clinical well-being carries an unacceptably high risk of relapse or catastrophic vascular events [1,5,6].

Pathogen isolation in SBO is notoriously difficult, and a high proportion of cases return sterile cultures due to prior antibiotic exposure [1,2,5]. When microbiological confirmation fails, empiric broad-spectrum antimicrobial regimens targeting *Pseudomonas aeruginosa* and *Staphylococcus aureus* must be maintained for prolonged periods, typically six to 20 weeks, with de-escalation strictly guided by normalization of inflammatory markers and regional soft-tissue resolution on serial scans [1,5,6]. In our patient, combining ceftazidime with ciprofloxacin delivered synergistic bactericidal activity alongside excellent osseous and central nervous system penetration, fully aligning with current pediatric consensus guidelines [2,5].

Simultaneously, therapeutic anticoagulation remains standard practice in managing septic dural venous sinus thrombosis, particularly within the cavernous sinus, to arrest thrombus propagation, promote recanalization, and mitigate long-term neurological deficits [6]. The transition from low-molecular-weight heparin to oral rivaroxaban syrup in this case reflects a shifting paradigm toward direct oral anticoagulants in pediatric medicine, offering a predictable, non-invasive outpatient alternative [6]. Our patient’s favorable recovery demonstrates that aggressive, targeted dual-antibiotic therapy, paired with structured anticoagulation, can optimize survival in pediatric central SBO, even when complicated by multisinus thrombosis. While the child achieved excellent systemic health and complete otological recovery at the three-month mark, the persistent, non-compensatory convergent strabismus underscores the real risk of lasting cranial nerve morbidity, necessitating long-term ophthalmological follow-up and potential corrective surgery [6,10].

## Conclusions

Central SBO compounded by extensive dural venous sinus thrombosis represents an exceptionally rare, life-threatening sequela of pediatric AOM. This case underscores that the acute onset of isolated strabismus or orbital symptoms in a child with a recent ear infection demands immediate advanced neuroimaging via high-resolution CT and contrast-enhanced MRI. Early detection of characteristic clival bone marrow alterations and cavernous sinus infiltration is critical to bypass hazardous diagnostic delays and confidently exclude skull base malignancies. Ultimately, an aggressive, multi-modal strategy combining prolonged targeted antimicrobial therapy with therapeutic anticoagulation remains vital to halting the infectious cascade and maximizing neurological recovery. While this single case report highlights critical diagnostic and therapeutic pathways, further studies are warranted, as these findings from an individual case may not universally apply to all similar clinical presentations.
